# *L*_3_-edge X-ray spectroscopy of rhodium and palladium compounds

**DOI:** 10.1107/S1600577524004673

**Published:** 2024-06-26

**Authors:** Hugo Alexander Suarez Orduz, Luca Bugarin, Sarina-Lena Heck, Paolo Dolcet, Maria Casapu, Jan-Dierk Grunwaldt, Pieter Glatzel

**Affiliations:** a ESRF – The European Synchrotron, 71 Avenue des Martyrs, 38000Grenoble, France; b Institute for Chemical Technology and Polymer Chemistry (ITCP) Karlsruhe Institute of Technology Engesserstr. 18/20 76131Karlsruhe Germany; c Ecole Doctorale de Physique Grenoble Alpes University 38400Saint-Martin-d’Hères France; d Institute of Catalysis Research and Technology (IKFT) Karlsruhe Institute of Technology 76344Eggenstein-Leopoldshafen Germany; University of Turin, Italy

**Keywords:** HERFD-XANES, valence orbitals, 4*d* transition metal, electronic structure, tender X-rays

## Abstract

*L*_3_-edge high-energy-resolution fluorescence-detection X-ray absorption near-edge structure (XANES) spectra for palladium and rhodium compounds are presented, with focus on their electronic structures. The data are compared with transmission XANES spectra recorded at the *K*-edge. A correlation between the absorption edge energy and the metal ion oxidation state is not observed. Despite the different filling of the 4*d* orbitals and different local coordination, the Rh and Pd compounds show remarkably similar spectral shapes. Calculation of the density of states and of the *L*_3_-XANES data reproduce the experimental results.

## Abstract 2

The *K* and *L*_3_X-ray absorption near-edge structures of Rh and Pd compounds are compared.

## 1. Introduction

X-ray spectroscopy studies to investigate the electronic structure and ligand environment of 4*d* transition metals are generally carried out at the *K*- or *L*-edges (Levin *et al.*, 2020 ▸; Zeeshan *et al.*, 2019 ▸; Gatla *et al.*, 2017 ▸). Within the dipole approximations for optically excited one-electron transitions, the *K*-edge and *L*-edge probe orbitals or unoccupied electron density of states with *p* (*l* = 1) and *d* (*l* = 2) character, respectively. The core hole lifetime broadening at the *K*-edge of Rh and Pd is around 6 eV while it is around 2.2 eV at the *L*_3_-edge (Krause & Oliver, 1979 ▸). Hence, the *K*-edge is ideally used for measurements of the extended X-ray absorption fine structure (EXAFS) while the *L*-edge allows directly probing the valence orbital electronic structure. The *L*-edges are located in the tender X-ray range, where the absorption of X-rays is strong. This makes *in situ* and *operando* studies challenging because the instruments need to be adapted to follow the reaction in vacuum conditions. This environment removes air, which otherwise attenuates the signal from the sample (Nowak *et al.*, 2020 ▸; Suarez Orduz *et al.*, 2024 ▸; Rovezzi *et al.*, 2020 ▸). Furthermore, the short penetration depth increases the apparent sample inhomogeneity at the incoming X-ray energy as only a very thin layer (sub-micrometre in the absorption white line) is probed. The Bragg angle of the crystal monochromator must be scanned over a larger range at lower energies decreasing the beam stability. However, more and more experimental setups are being conceived that overcome such limitations (Vitova *et al.*, 2013 ▸; Kavčič *et al.*, 2016 ▸; Robba *et al.*, 2017 ▸; Shakouri *et al.*, 2020 ▸; Suarez Orduz *et al.*, 2024 ▸). The spectral broadening can be further reduced by employing an X-ray spectrometer based on perfect crystal Bragg optics where the energy bandwidth in the X-ray detection is reduced by two orders of magnitude as compared with solid state detectors. High-energy-resolution fluorescence-detected X-ray absorption spectroscopy (HERFD-XAS) almost completely eliminates any background with a spectral resolution that is limited by the lifetime broadening of the final state of radiative transition (Glatzel *et al.*, 2009 ▸; Glatzel & Bergmann, 2005 ▸; Hämäläinen *et al.*, 1991 ▸). High-energy-resolution fluorescence-detected X-ray absorption near-edge structure (HERFD-XANES) offers improved energy resolution over traditional XANES methods by recording the *L*α_1_ emission line with an instrumental energy bandwidth below the 2*p*_3/2_ core hole lifetime broadening.

The significance of rhodium and palladium compounds has surged, particularly in domains like catalysis, fuel cells and electronic devices (Sharma *et al.*, 2019 ▸). Their notable catalytic efficiency is widely utilized across various sectors, including pharmaceutical synthesis, plastics manufacturing and emission control for clean air (Pawlak *et al.*, 2014 ▸; Grushin, 2010 ▸; Fabry & Rueping, 2016 ▸). These elements are distinguished by their poisoning and corrosion resistance, electrical conductivity and thermal stability, making them indispensable in electronics (Narayan *et al.*, 2019 ▸; Iavicoli & Leso, 2015 ▸; Hubicki & Leszczyńska, 2005 ▸). Pd is one of the central elements for methane and hydro­carbon removal, which will also be important in future considering biogas and power-to-gas processes for energy storage (Kalz *et al.*, 2017 ▸). In addition, Rh is one of the central elements for NO_*x*_ conversion in three-way catalysts. In fuel cell technology, Pd catalysts are particularly noted for maintaining long-term activity in oxidation reactions (Zhang *et al.*, 2011 ▸; Akhairi & Kamarudin, 2016 ▸). Consequently, exploring the electronic structure of these compounds is crucial for understanding their physical and chemical properties. Employing X-ray spectroscopy in conjunction with *ab initio* calculations has been instrumental in studying these properties, enhancing our comprehension of the connection between electronic structure and chemical functionality (Garino *et al.*, 2014 ▸; Mattioli *et al.*, 2015 ▸).

HERFD-XANES has become a pivotal tool for in-depth analysis of local electronic structures around absorbing atoms (Glatzel *et al.*, 2009 ▸; Shimizu, Kamiya *et al.*, 2012 ▸; Shimizu, Oda *et al.*, 2012 ▸). HERFD-XANES enables a more profound investigation into the structure and composition of materials through superior spectral resolution and reduced background signal (Rovezzi & Glatzel, 2014 ▸; Glatzel & Bergmann, 2005 ▸; Glatzel *et al.*, 2013 ▸). Analysis of the *L*_3_-edge in 4*d* transition metals, in particular, provides valuable insights into electronic structures and local coordination, probing empty *d* orbitals via the dipole allowed 2*p* to 4*d* transition. The strong spin–orbit interaction of the 2*p* electron splits the 2*p*^5^4*d*^*n*+1^ excited state into *L*_2_ and *L*_3_ components that show different spectral shapes due to the Δ*j* = 0, ±1 selection rule (Kasper *et al.*, 2018 ▸). It was shown that density functional theory (DFT) calculations are suitable to model the *L*_3_-edges of 4*d* transition metals to sufficient accuracy in order to extract valuable information on the local coordination (Bunău & Joly, 2009 ▸; Svyazhin *et al.*, 2022 ▸; Mistonov *et al.*, 2018 ▸).

To pave a basis to use HERFD-XANES at the *L*_3_-edges combined with corresponding spectra at the *K*-edges for *in situ* and *operando* studies in future, we recorded *K*-edge XANES in transmission mode and *L*_3_-edge HERFD-XANES across a range of palladium and rhodium compounds. This investigation includes a diverse array of samples: Rh foil, RhCl_3_, RhO_2_, Rh_2_O_3_ for rhodium and Pd foil, Pd(OAc)_2_, Pd(OH)_2_, PdO, Pd(acac)_2_ for palladium, encompassing various oxidation states and chemical environments. We compare the electronic structures of these compounds, as revealed by XAS at the*K*-edge and HERFD-XANES *L*_3_-edge techniques. The study extends to a detailed comparison between HERFD-XANES *L*_3_-edge data and *L*_3_-edge simulations for metallic Rh, RhO_2_, Rh_2_O_3_, metallic Pd and PdO. This approach enables a deeper exploration into how the oxidation states and ligand environments influence the absorption edge locations and offers a nuanced view of the electronic structures, with a specific focus on the 4*d* orbital contributions.

Through this extensive analysis, we aim to elucidate how the variations in electronic structures are influenced by different chemical states and local environments. The enhanced resolution of HERFD XANES at the *L*_3_-edge, in combination with the predictive insights from DFT simulations, allows for a more detailed understanding of the electronic structures of these diverse palladium and rhodium compounds. The findings from this comparative study contribute to our knowledge of the electronic properties of these materials, which are crucial in catalysis and electronic device manufacturing.

## 2. Experimental section

HERFD-XANES measurements were carried out using the tender X-ray emission spectrometer (TEXS) at beamline ID26 of the ESRF (Rovezzi *et al.*, 2020 ▸). The incident energy was selected by means of the (111) reflection of a cryogenically cooled Si double-crystal monochromator. The total flux was 5 × 10^13^ photons s^−1^ using the fundamental harmonic. The beam size (full width at half-maximum) was 250 µm × 40 µm (horizontal × vertical). Higher harmonics were suppressed by two Si mirrors in total reflection at 5 mrad angle of incidence. The flux of the incoming monochromatic X-rays was recorded by detecting X-ray scattering from a Kapton film on a photodiode. Metallic Rh and Pd foils were used for the calibration of the incident beam energy. In the TEXS chamber, five cylindrical Si(111) Johansson crystal analyzers were used. The fluorescence intensity was recorded using a gas-proportional counter. The HERFD-XANES spectra were recorded by monitoring the fluorescence intensity at the maximum of the Rh *L*α_1_ (2696.80 eV, Bragg angle 47.15°) and Pd *L*α_1_ (2837.80 eV, Bragg angle 44.16°) emission lines. The samples were checked for radiation damage by recording ten consecutive XAS scans (on-the-fly data acquisition) of 30 s each. It was concluded that none of the compounds were radiation sensitive.

The absorption length at the *L*_3_-edge of a 4*d* transition metal oxide is only a few micrometres above the resonance and below a micrometre at the resonance energy. The particle size for samples where over-absorption can be neglected should be a few hundred nanometres for an oxide, which is very difficult to achieve. Therefore, we refrain from attempting to correct for over-absorption in the thick sample limit and consider the spectral distortions due to over-absorption in the analysis. However, we estimated the effect of the spectral absorption using our code (Bianchini & Glatzel, 2012 ▸). The code uses a simple way to estimate the spectral distortion owing to of incident-beam self-absorption. First, the program determines the fluorescence counts using the tabulated cross section. Then the calculation is repeated using a scaled cross section, *i.e.* the tabulated value multiplied by a given scaling factor. The tabulated values used in the program correspond to the edge jump and a scaling by five that would thus simulate a white line with maximum five times stronger intensity than the edge-jump normalized spectrum (Bianchini & Glatzel, 2012 ▸). We found that the white-line intensity maximum is suppressed by a factor of two assuming that it has a five times higher cross-section than the edge jump. We estimate that this would introduce a relative error in the determination of the edge energy of approximately 0.2 eV between a scan free of over-absorption and a distorted scan. As all recorded spectra are similarly affected by over-absorption, we estimate the relative error when comparing between the compounds presented here to be less than 0.1 eV.

The HERFD-XANES spectra at the *L*_3_-edge of Rh and Pd were simulated using the *FDMNES* code (Joly *et al.*, 2009 ▸). This code allows the calculation of the density of states (DOS) in relation to the X-ray absorption process. Simulations were performed for an atomic cluster with radii from 3 Å to 9 Å. The local density approximation was used to calculate the potential. The reduced lifetime broadening in the HERFD-XANES spectra was considered by adjusting the parameters for spectral broadening. The input parameters are provided in the supporting information.

Transmission-mode XAS measurements at the Pd (24350 eV) and Rh (23220 eV) *K*-edges were conducted using a Si(111) double-crystal monochromator in continuous scanning mode at the ESRF beamline BM23. Energy ranges were 24258–25253 eV for Pd and 23135–24232 eV for Rh. Harmonic rejection was achieved by flat mirrors with Pt coating at 2.8 mrad incident angle. Simultaneous XANES spectra of Pd/Rh foils ensured precise energy calibration. Ionization chambers (I0, I1) measured incident and transmitted X-ray intensities, with a third chamber for Pd foil reference XANES measurements.

Pellets of diameter 5 mm were obtained by mixing the ground powder of Pd and Rh compounds with cellulose, which served as a binder. The components in the mixture were weighed to achieve a consistent 1% weight, and ground until the mixture was uniform. Samples were then pressed in pellets, which were used for both HERFD-XANES measurements at the *L*_3_-edge and XAS transmission measurements at the *K*-edge. X-ray diffraction patterns of Pd(OAc)_2_, Pd(acac)_2_, PdO, Rh_2_O_3_, RhO_2_ and RhCl_3_ were obtained at the ID31 beamline of the ESRF using an X-ray wavelength of 0.164 Å. The data were recorded with a two-dimensional Pilatus 2M CdTe detector. Details are provided in the supporting information.

## 3. Results and discussion

### 3.1. Spectral normalization of HERFD-XANES at the Pd/Rh *L*_3_-edge

The *L*_3_-edge of Rh/Pd is dominated by a resonant 2*p* to 4*d* transition resulting in a 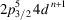 excited state electron configuration. The edge jump defined as the onset of transitions into the continuum is at higher energies than the resonance but the precise value is difficult to determine. The separation into resonant and continuum excitations is a simplifying concept and there is no strict definition on where this edge jump energy should be. The short absorption length and longer wavelength in the tender X-ray range furthermore exacerbate experimental artifacts that one may encounter in X-ray spectroscopy (Northrup *et al.*, 2016 ▸). The monochromator crystals are scanned through a larger angular range with the beam possibly moving across the sample and scattering foil used for flux measurement. The first micrometre of the sample surfaces appears more inhomogeneous than a volume with thickness of tens of micrometres. Thus, even pressed pellets may appear very inhomogeneous to the X-ray beam that penetrates only a few hundred nanometres into the sample. The situation is worse for *operando* measurements in heterogeneous catalysis where the sample is prepared as a sieved powder to allow the gas flow through the catalyst bed. Here, furthermore, the gas flow as well as heating and cooling down may move the sample and consequently create artifacts in the spectra.

We conclude that a robust normalization to the edge jump is very difficult to achieve for many samples in the tender X-ray range. We, therefore, additionally investigated whether the spectra can be normalized to the spectral area. The area normalization follows the previously reported *f*-sum rule (Johnson, 1974 ▸), ensuring that the system’s fundamental properties are reflected. This enables the identification of genuine differences in sample structure while sidelining artifact effects as much as possible. Therefore, we find that area normalization is a suitable approach that we followed in the data that are presented here. The energy ranges that were used for the spectral area normalization are 3159.98–3195.00 eV for Pd and 2990.00–3036.00 eV for Rh.

### 3.2. *L*_3_-edge HERFD and *K*-edge transmission XANES

**Figure 1:**
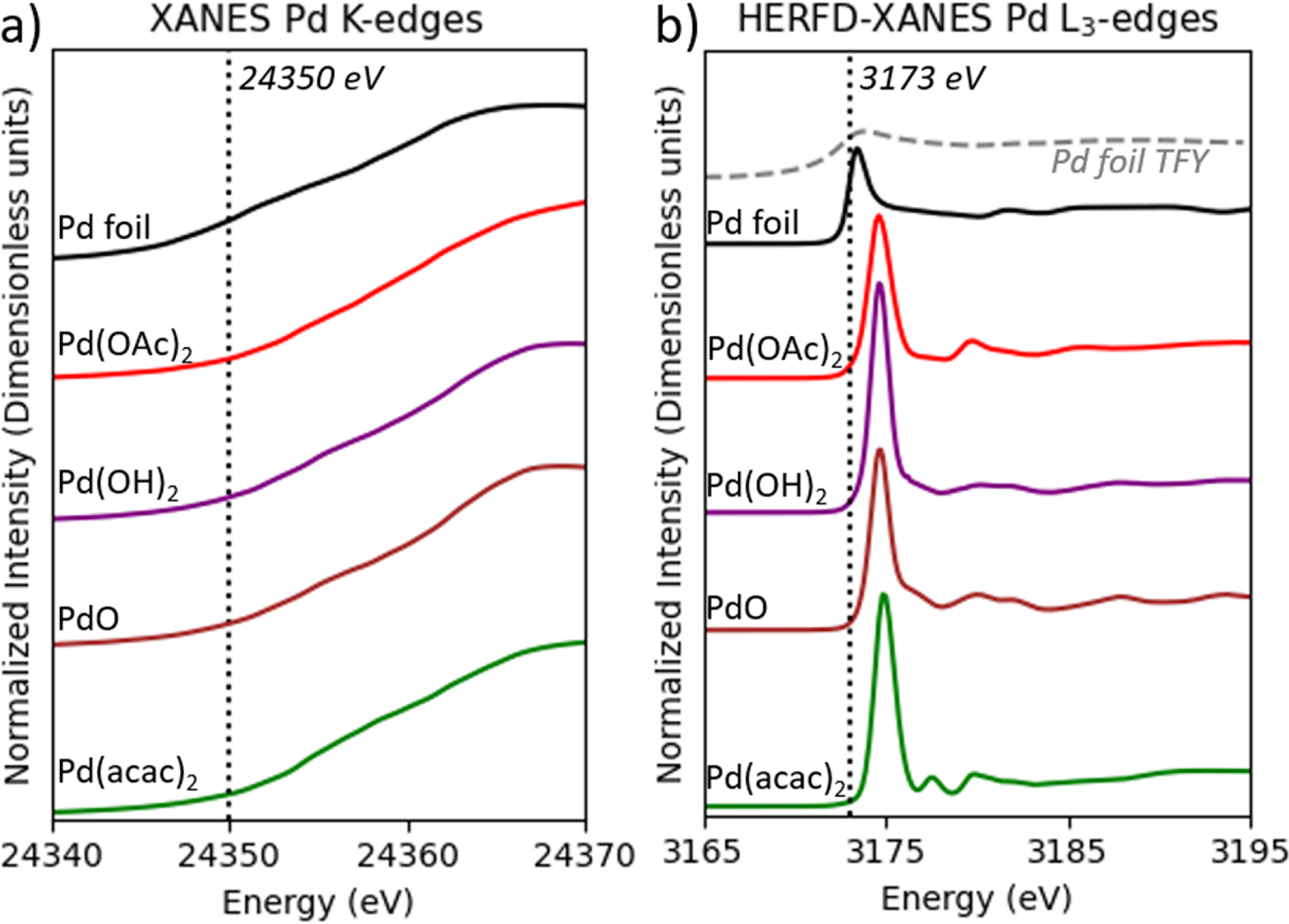
Pd *K*-edge transmission XANES and Pd *L*_3_-edge HERFD-XANES spectra for various Pd compounds. The dotted black lines indicate the FIP of the Pd metal absorption edge: Pd metallic, Pd(OAc)_2_, Pd(OH)_2_, PdO, Pd(acac)_2_.

Figs. 1 ▸ and 2 ▸ show the X-ray absorption near-edge structure at the *K*- and *L*_3_-edges for various Pd and Rh compounds. All graphs cover about 20 eV above the Fermi energy that include the lowest unoccupied molecular orbitals that are most strongly modified by the local coordination. We exclude the EXAFS region in our analysis. The *K*-edge XANES region for Pd and Rh compounds lacks distinct features over the 20 eV range due to the large core hole lifetime broadening. The lack of spectral features and the fact that orbitals with *p* orbital moment are probed strongly limits the information that can be obtained in the *K*-edge XANES region (Svyazhin *et al.*, 2022 ▸; Timoshenko & Roldan Cuenya, 2021 ▸; Wang *et al.*, 2023 ▸). The total fluorescence yield XANES data are also shown for the metal foils to illustrate the line sharpening effect in HERFD-XANES. These aspects were demonstrated in previous findings. For instance, Svyazhin *et al.* (2022 ▸) provided a comprehensive analysis of the Mo *L*_3_-edge HERFD-XANES data, similarly detailing how HERFD-XANES highlights spectroscopic changes as a function of the chemical environment and coordination in transition metals.

We determined the first inflection point (FIP) of the XANES spectra for Pd and Rh, detailed in Table 1 ▸. Our findings confirm that the molecular symmetry influences the location of the FIP. Table 1 ▸ illustrates the characteristics of the square planar geometry of Pd(II) with four surrounding atoms, contrasted by octahedral coordination of Rh(III) and Rh(IV) surrounded by six atoms. Generally, the same trends were observed at *K*- and *L*_3_-edges, with lower-energy FIPs in metallic Pd and Rh compared with their oxidized forms. Increased oxidation states enhance the positive charge of the metal, strengthening metal–ligand bonds and altering orbital energies. These changes lead to greater excitation energy, shifting the absorption edges to higher energies due to reduced electronic shielding (Eisenberger & Kincaid, 1978 ▸). In ligand-bound complexes (entries 2–5, 7–9), FIP energy rises with ligand field strength, signaling stronger metal–ligand covalency. This effect arises from the electron distribution within the molecular orbitals of the complex, optimizing the energy of the system (Wulfsberg, 2000 ▸; Sasaki & Kiuchi, 1981 ▸). Shorter metal–ligand distances indicate tighter chemical bonds, thus elevating absorption-edge energies. A higher electronegativity of the neighboring atom should result as well in a shift of the edge position to higher energy (Pantelouris *et al.*, 2004 ▸). However, the crystal field splitting adjusts as well with metal–ligand bond length, proving that both the covalency and the structural configuration influence the spectroscopic outcome (Svyazhin *et al.*, 2022 ▸; Bunker, 2010 ▸).

The energy of the absorption edge of Rh(IV) oxide is lower than that of Rh(III) oxide, which is in contrast to what is observed at the *K*-edges of the 3*d* transition metals for different oxidation states. Similar trends were found for other 4*d* and 5*d* transition metals in previous investigations (Farges, 2009 ▸; Tromp *et al.*, 2007 ▸; Pantelouris *et al.*, 2004 ▸). Therefore, these findings suggest that caution should be exercised when directly correlating the *L*_3_-edge position with the oxidation state. With respect to the ligand electronegativity, the position of both the *K*- and *L*_3_-absorption edges is at lower energies for RhCl_3_ in comparison with Rh_2_O_3_, which is in agreement with analogous literature data (Pantelouris *et al.*, 2004 ▸; Tromp *et al.*, 2007 ▸). For the palladium compound series, the highest absorption energy of the *K*-edge was measured for Pd(acac)_2_ and the lowest for Pd(OAc)_2_, a trend followed also at the *L*_3_-edge. On the other hand, a sharp and symmetrical white line may indicate a more uniform environment around palladium, while a broader and asymmetrical peak could signal greater complexity or variation in the local structure (Chassé *et al.*, 2018 ▸; Henderson *et al.*, 2014 ▸).

To deepen the understanding of the influence of noble metal local structure and electronic state on the observed spectroscopic properties, five compounds were selected: metallic Pd, PdO, metallic Rh, RhO_2_ and Rh_2_O_3_ for a comparative analysis using HERFD-XANES spectra and DFT simulations at the *L*_3_-edge. This selection covers different coordination and oxidation states, allowing a detailed exploration of their impact on the spectroscopic signals. The unit cells shown in Fig. 3 ▸, with Pd atoms represented by silver spheres and Rh by gray spheres, highlight the structural differences: both metallic Pd and Rh exhibit a cubic structure and octahedral geometry, PdO is characterized by its tetragonal configuration and square-planar coordination, while RhO_2_ and Rh_2_O_3_, despite sharing octahedral coordination, differ in their crystalline symmetry, being tetragonal and trigonal, respectively.

**Figure 2:**
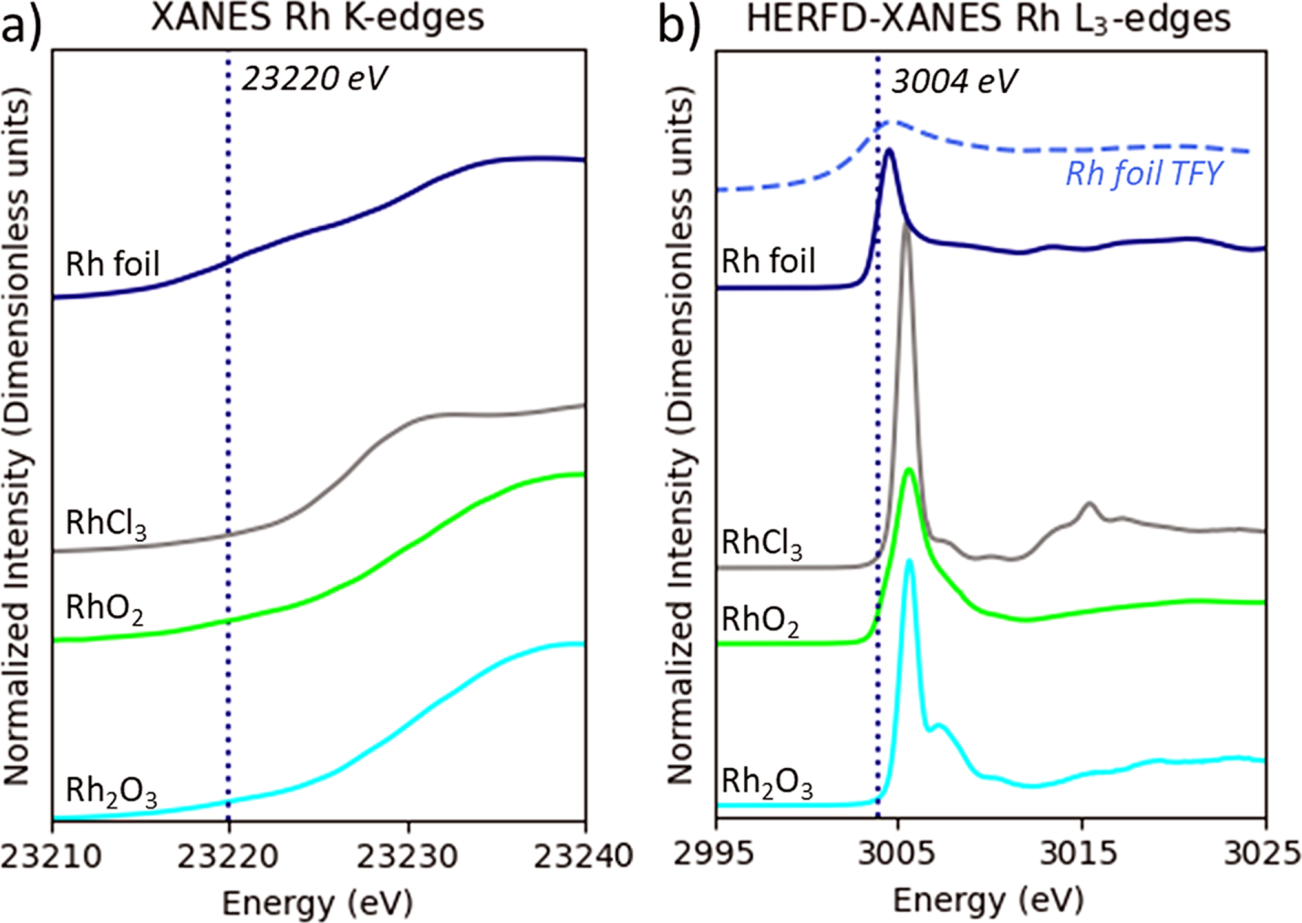
Rh *K*-edge transmission XANES and Rh *L*_3_-edge HERFD-XANES spectra for various Rh compounds. The dotted black lines indicate the FIP energy of the Rh metal absorption edge: Rh metallic, RhCl_3_, RhO_2_, Rh_2_O_3_

**Figure 3:**
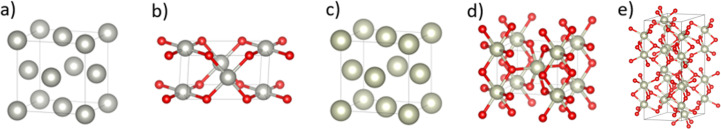
Unit cells of metallic Pd (*a*), PdO (*b*), metallic Rh (*c*), RhO_2_ (*d*) and Rh_2_O_3_ (*e*) structures.

**Figure 4:**
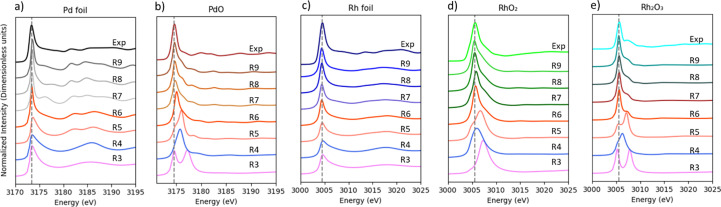
Experimental and simulated HERFD-XANES spectra at the *L*_3_-edge for different cluster sizes (*R* = 3–9 Å).

**Figure 5:**
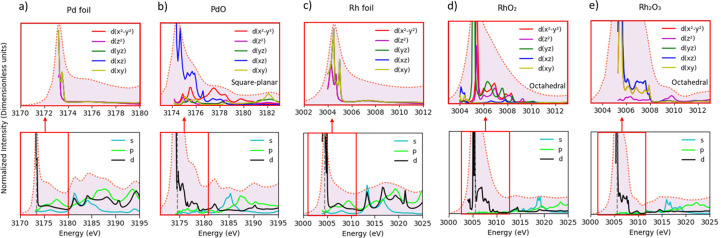
Angular momentum projected density of states (*s*-, *p*- and *d*-DOS) for simulations of clusters with a radius of 9 Å, and the decomposition of *d* orbitals into *d*_*x*^2^−*y*^2^_, *d*_*z*^2^_, *d*_*xz*_, *d*_*yz*_ and *d*_*xy*_ transitions for samples of Pd foil, PdO, Rh foil, Rh_2_O_3_ and RhO_2_.

Calculated spectra obtained by using the crystallographic information files (CIFs, as detailed in the supporting information) are shown in Fig. 4 ▸ and the angular orbital momentum projected DOS in Fig. 5 ▸. Fig. 4 ▸ illustrates how the increase of the noble metal cluster size significantly alters the spectral shape until it closely aligns with the experimental results. Convergence of the simulation is typically achieved for a cluster radius of 9 Å in most cases while a smaller radius occasionally displayed splitting of peaks. In agreement with Svyazhin *et al.* (2022 ▸), we found that the DFT calculations considering one-electron transitions and a full core hole provide a suitable tool for correlating the spectral shape with an atomic structural motif. Comparison of the data reported in Figs. 2 ▸, 4 ▸ and 5 ▸ illustrates how the onset of the *p*-DOS, as measured at the *K*-edge, is at higher energy than the resonance in the *d*-DOS, as measured at the *L*_3_-edge. The metals show a strong slightly asymmetric resonance while the oxides develop a distinct shoulder on the high energy side of the main resonance. The main resonance is almost pure *d*-DOS while the shoulder arises from DOS that is dominant *d* in character but has *s* and *p* admixture. This behavior is remarkably similar for PdO and the Rh oxides even though Pd is planar, four-coordinated and Rh is six-coordinated. The *d* orbitals pointing out of the [PdO_4_] plane are populated and only the in-plane orbitals in Pd(II) can receive an electron from the 2*p* shell. There are two orientations of the [PdO4] planes in the crystal structure and the decomposition of the *d*-DOS into its *d*_*x*^2^–*y*^2^_, *d*_*z*^2^_, *d*_*xz*_, *d*_*yz*_ and *d*_*xy*_ components does not show a crystal field splitting. Orbital mixing results in a large energy spread of the components. PdO could be considered to exhibit high-spin characteristics due to its complex magnetic properties (Sirajuddeen *et al.*, 2020 ▸). However, spin–orbit effects significantly mix the states of PdO, altering the electronic structure and spin configuration of Pd(II). These effects are important for understanding the electronic and magnetic properties of Pd in square planar symmetry and the impact these have when Pd interacts with other ligands (Almeida *et al.*, 2018 ▸). Rh(III, 4*d*^6^) and Rh(IV, 4*d*^5^) spectra are expected to show a crystal field splitting but only one strong resonance feature similar to Pd was observed. Svyazhin *et al.* (2022 ▸) clearly resolved the transitions to *t*_2*g*_ and *e*_*g*_ orbitals in Mo perovskite compounds but a less distinct structure was found for the transitions into *e*_*g*_ for Mo oxides (broader peaks). Calculations for smaller cluster sizes for Rh_2_O_3_ yield two crystal field split peaks. Increasing the cluster size creates orbitals that are mixed and extended over several coordination spheres. Overall, the *d*-DOS is qualitatively similar for the Pd and Rh oxides even though their formal 4*d* orbital population is very different. It appears that the orbital mixing for all but the lowest energy 4*d* orbital renders a rather inaccurate crystal field picture of the valence orbitals. These observations confirm that the 4*d* orbital localization and thus excitonic character of the 2*p*^5^4*d*^*n*+1^ excited state is very different between 3*d* and 4*d* transition metals as orbital mixing shapes the 4*d* orbital considerable more.

## 4. Conclusions

More and more experimental stations worldwide offer tender X-ray spectroscopy and some feature X-ray emission spectrometers that enable HERFD-XANES measurements. Furthermore, *in situ* and *operando* cells have been developed thus offering a large range of tools for studies in materials science. The information that can be obtained based on EXAFS data collected at the *K*-edge and HERFD-XANES analysis of the *L*_3_-edge is complementary, and ideally both experiments should be conducted. This may become more accessible in the future as many beamlines make efforts to cover broad energy ranges.

In this context, this study shows that the spectroscopic properties of Pd and Rh compounds are influenced by coordination geometry and structural symmetry. XAS measurements at the *K*- and *L*_3_-edges combined with *FDMNES* simulations of the HERFD-XANES spectra directly correlate the contribution of orbitals, bonding and atomic structures to the experimental spectral shapes. The obtained results emphasize how variations in oxidation state, local symmetry and metal–ligand covalency, influenced by ligand field strength, impact metal–ligand distances and the position especially of the *L*_3_-absorption edge. The high energy resolution of HERFD-XANES data and the pronounced spectral changes of the *L*_3_-edge of 4*d* transition metals provide favorable conditions for studying strongly heterogeneous systems that contain the absorber elements in several different chemical states, as often encountered during *in situ* and *operando* investigations. This is currently only rarely exploited and the emerging possibilities will be explored in the coming years.

## 5. Related literature

The following reference, not cited in the main body of the paper, has been cited in the supporting information: Amidani *et al.* (2021 ▸).

## Supplementary Material

Sections S1 to S3, including Figs. S1 and S1 and Table S1. DOI: 10.1107/S1600577524004673/uc5003sup1.pdf

## Figures and Tables

**Table 1:** Edge energies of measured compounds

Compound	Oxidation state and formal 4*d* orbitals population	Distance metal–ligand (Å)	Coordination geometry	FIP *K*-edge white line [+24300 eV]	FIP *L*_3_-edge white line [+3100 eV]	Reference
Pd		2.751 (metal–metal)	Octahedral	45.4	73.0	(Owen & Yates, 1933 ▸)
Pd(OAc)_2_	II, 4*d*^8^	1.992	Square planar	53.2	74.1	(Skapski & Smart, 1970 ▸)
Pd(OH)_2_	II, 4*d*^8^	∼2	Square planar	53.9	74.2	(Troitskii *et al.*, 1995 ▸)
PdO	II, 4*d*^8^	2.018	Square-planar	54.1	74.2	(Waser *et al.*, 1953 ▸)
Pd(acac)_2_	II, 4*d*^8^	1.9835	Square-planar	54.4	74.4	(Hamid *et al.*, 2005 ▸)

				[+23200 eV]	[+3000 eV]	
Rh		2.539 (metal–metal)	Octahedral	15.1	3.7	(Moshopoulou *et al.*, 2006 ▸)
RhCl_3_	III, 4*d*^6^	2.292 to 2.31	Octahedral	22.2	4.8	(Bärnighausen & Handa, 1964 ▸)
RhO_2_	IV, 4*d*^5^	1.928	Octahedral	23.7	4.8	(Shannon, 1968 ▸)
Rh_2_O_3_	III, 4*d*^6^	2.042 to 2.071	Octahedral	25.4	5.0	(Kim *et al.*, 2021 ▸)
