# Supplementary material for: L3-edge X-ray spectroscopy of rhodium and palladium compounds
Source: J Synchrotron Radiat. 2024 Jun 26;31(Pt 4):733–40. doi: 10.1107/S1600577524004673 (PMC11226176; doi:10.1107/S1600577524004673)
Supplement: Supplementary file 1 [file s-31-00733-sup1.pdf]

JOURNAL OF SYNCHROTRON RADIATION

Volume 31 (2024)

Supporting information for article:

L_3-edge X-ray spectroscopy of rhodium and palladium compounds

Hugo Alexander Suarez Orduz, Luca Bugarin, Sarina-Lena Heck, Paolo Dolcet, Maria Casapu, Jan-Dierk Grunwaldt and Pieter Glatzel

S1. Simulation of HERFD-XANES Spectra

HERFD XANES spectra simulations were carried out using the "Finite Difference Method Near Edge Structure" (FDMNES) software, specifically designed to model the near-edge absorption structure of X-rays. This program employs a mono-electronic approach that combines multiple scattering theory with the finite difference method, all within a fully relativistic context that includes spin-orbit interaction (Joly *et al.*, 2009). Simulations of HERFD-XANES spectra at the L_3 absorption edge for Pd, PdO, Rh, RhO_2, and Rh_2O_3 were carried out, applying the following general parameters:

- Energy range: -1, 0.1, 5, 0.25, 20, 0.5, 50. This setting allows for a finer energy step near the absorption edge and a larger step away from it, optimizing resolution in areas of interest and computational efficiency in less critical regions.
- L_3-Edge: Specifies the simulation at the L3 absorption edge, focusing the study on the electronic transitions relevant to these materials.
- Spgroup: Defines the space group of each compound, an important parameter for modeling the crystalline structure and symmetries of the material.
- SCF (Self-Consistent Field): Includes self-consistent calculations of the electronic structure, iteratively adjusting wave functions and energies to obtain a stable solution that satisfies the Hartree-Fock or DFT equations.
- Relativism: Incorporates relativistic effects in the simulations, which are significant for heavy elements or for high-precision features.
- Spinorbit: Considers the spin-orbit interaction, essential for understanding electronic transitions at L absorption edges, where these effects are pronounced.
- Excited: Simulates the absorbing atom in an excited state, allowing the study of transitions and electronic changes during absorption.
- Density: Calculates and uses the s, p, and d density of states, offering a detailed view of the electronic distribution in the material.
- Green: Applies the Green's function to solve the Schrödinger equation, using the "muffin-tin" approximation for the potential calculation.

To adapt a simulation to HERFD XANES instead of conventional XANES, it is essential to precisely adjust the convolution and energy level broadening parameters. Key parameters include "Convolution", "Gamma_hole", and "Gamma_max", each playing significant roles in this process. Convolution simulates the broadening of spectral lines caused by physical effects such as the limited lifetime of excited states and interaction with photons, which helps to replicate the spectral profile observed in experiments.

- "Gamma_hole" Core level width (Γ_{Hole}) in eV. It determines the width of the convolution for the broadening of energy levels due to the creation of a hole in the core level after photon absorption. It is set in most of the cases around to "0.5" eV, reflecting how the core state's lifetime and electron-electron interactions influence energy absorption. Adjusting this value allows for a more accurate simulation of the impact of core hole creation in the HERFD XANES simulation.
- "Gamma_max", with a set value of "3.5", indicates the maximum broadening applied in the simulation, limiting the broadening of excited states and the core hole.

We adjusted the Gamma_hole and Gamma_max values to align with the experimental spectrum. These values are based on Gamma_hole and Gamma_max values reported for HERDF-XANES spectra of transition metals from the literature (Svyazhin *et al.*, 2021; Amidani *et al.*, 2021). In the convolution of XANES spectra, the EFermi represents the energetic reference point from which electronic transitions are determined. The variable Ecent is crucial as it defines the center of the resonance of the arctangent function, reflecting the energy where the main transition occurs. On the other hand, Elarg controls the width of the arctangent function, which is important for describing how the broadening of the spectral features varies with energy, allowing for a better match between simulated and experimental spectra.

Pd foil input R9

Filout
Pd-foil-13Apr-R7
Range
-1. 0.1 5. 0.25 20. 0.5 50.

Edge
L3 !***
!Energpho
Spgroup
Fm-3m
SCF
Relativism
Spinorbit
Excited
Density
Green
Radius
9
Crystal !***
3.957066 3.957066 3.957066 90 90 90
46 0.00000000 0.00000000 0.00000000
46 0.00000000 0.50000000 0.50000000
46 0.50000000 0.00000000 0.50000000
46 0.50000000 0.50000000 0.00000000
Convolution
Gamma_hole
0.6
Gamma_max
3.5
Estart
-20
End

PdO input R9

Filout
PdO-13Apr-R7
Range
-1. 0.1 5. 0.25 20. 0.5 50.
Edge
L3 !***
!Energpho
SCF
Spgroup
131
Relativism
Spinorbit
Excited
Density
Density_all
Green
Eimag
Radius
9
Crystal !***
3.06000 3.06000 5.37200 90 90 90
46 0.500000 0.000000 0.000000
8 0.000000 0.000000 0.250000

Convolution
E_cut
-0.79011
Ecent
30
Elarg
10
Gamma_hole
0.9
Gamma_max
3.5
Estart
-20
End

Rh foil input R9

Filout
Rhfoil-13Apr-R7
Range
-1. 0.1 5. 0.25 20. 0.5 50.
Edge
L3 !***
!Energpho
Spgroup
Fm-3m
SCF
Relativism
Spinorbit
Excited
Density_all
Green
Eimag
Radius
9
Crystal !***
3.80340 3.80340 3.80340 90 90 90
45 0.00000000 0.00000000 0.00000000

Convolution
E_cut
-0.1
Ecent
2
Elarg
10
Gamma_hole
1
Gamma_max
3.5
Estart
-20
End

RhO_2 input R9

Filout
RhO2-13Apr-R7
Range
-1. 0.1 5. 0.25 20. 0.5 50.
Edge
L3 !***
!Energpho
SCF
Relativism
Spinorbit
Excited
Density
Density_all
Green
Eimag
Radius
9
Crystal !***
4.4862 4.4862 3.0884 90 90 90
45 0.000000 0.000000 0.000000
45 0.500000 0.500000 0.500000
8 0.306730 0.306730 0.000000
8 0.806730 0.193270 0.500000
8 0.193270 0.806730 0.500000
8 0.693270 0.693270 0.000000

Convolution
E_cut
-0.1
Ecent
5
Elarg
10
Gamma_hole
1
Gamma_max
3.5

Estart
-20
End

Rh_2O_3 input R9

Filout
Rh2O3-13Apr-R7
Range
-1. 0.1 5. 0.25 20. 0.5 50.
Edge
L3 !***
!Energpho
SCF
Relativism
Spinorbit
Excited
Density

```
Density_all
Green
Eimag
Radius
9
Crystal !***
5.14322   5.143220   13.887490   90.000000   90.000000
          120.000000

45   0.666670   0.333330   0.182520
45   0.333330   0.666670   0.317480
45   0.666670   0.333330   0.484150
45   0.000000   0.000000   0.349180
45   0.333330   0.666670   0.515850
45   0.000000   0.000000   0.650820
45   0.333330   0.666670   0.817480
45   0.666670   0.333330   0.682520
45   0.000000   0.000000   0.849180
45   0.666670   0.333330   0.984150
45   0.000000   0.000000   0.150820
45   0.333330   0.666670   0.015850
8    0.633200   0.666670   0.416670
8    0.333330   0.966540   0.416670
8    0.033460   0.366800   0.416670
8    0.700130   0.000000   0.250000
8    0.000000   0.700130   0.250000
8    0.299870   0.299870   0.250000
8    0.299870   0.000000   0.750000
8    0.000000   0.299870   0.750000
8    0.700130   0.700130   0.750000
8    0.366800   0.333330   0.583330
8    0.666670   0.033460   0.583330
8    0.966540   0.633200   0.583330
8    0.966540   0.333330   0.083330
8    0.666670   0.633200   0.083330
8    0.366800   0.033460   0.083330
8    0.033460   0.666670   0.916670
8    0.333330   0.366800   0.916670
8    0.633200   0.966540   0.916670

Convolution
E_cut
-0.839
Ecent
30
Elarg
30
Gamma_hole
0.7
Gamma_max
3.5
Estart
  -20
End
```

S2. Pd and Rh reference compounds

Table S1 Reference compounds for Pd and Rh with their CIF file codes and supplier information.

Order	Chemical Name	Supplier	CIF file
1	Pd	Goodfellow	ICDS Code 52251
2	$Pd(OAc)_2$	Strem	COD code 2014232
3	$Pd(OH)_2$	Sigma Aldrich	No CIF file
4	PdO	Sigma Aldrich	ICDS Code 24692
5	$Pd(acac)_2$	Sigma-Aldrich	COD code 2207229
6	Rh	goodfellow	ICDS 171677
7	$RhCl_3$	Abcr	ICDS Code 25764
8	RhO_2	Abcr	ICDS Code 28498
9	Rh_2O_3	Abcr	ICDS Code 113813

S3. XRD diffractograms of the Pd and Rh reference compounds

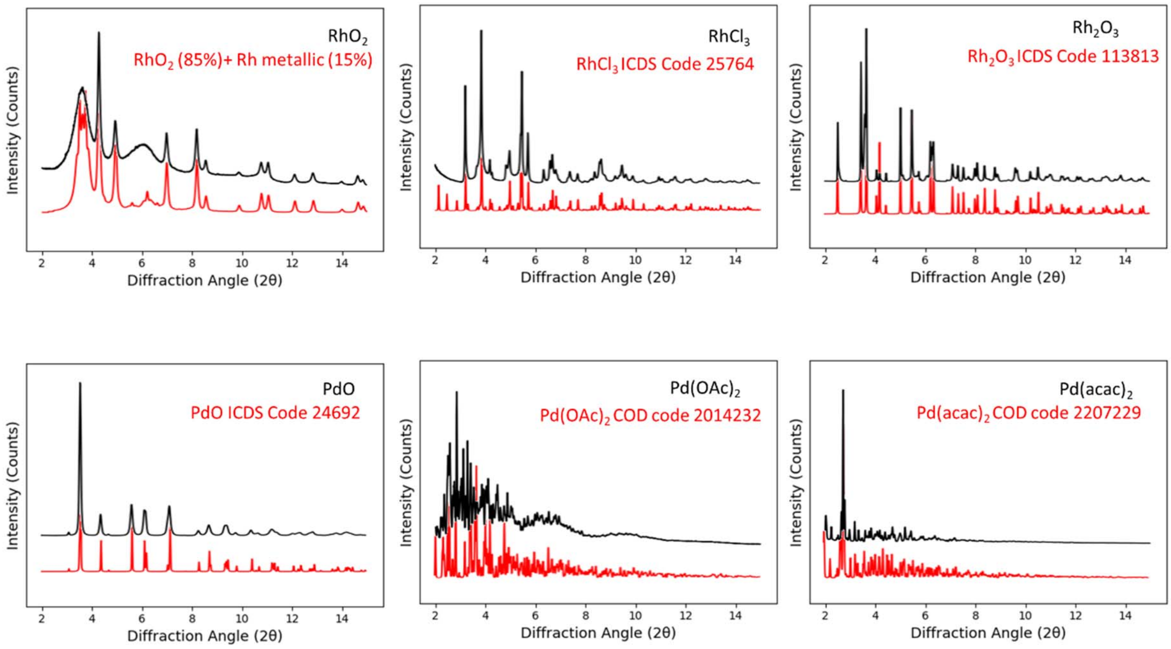

Figure S1 XRD diffractograms obtained for Pd(OAc)$_2$, Pd(acac)$_2$, PdO, Rh_2O_3, RhO_2, and $RhCl_3$ (wavelength of 0.164 Å). Black lines represent experimental data, while red lines depict the characteristic patterns as derived from the ICSD database

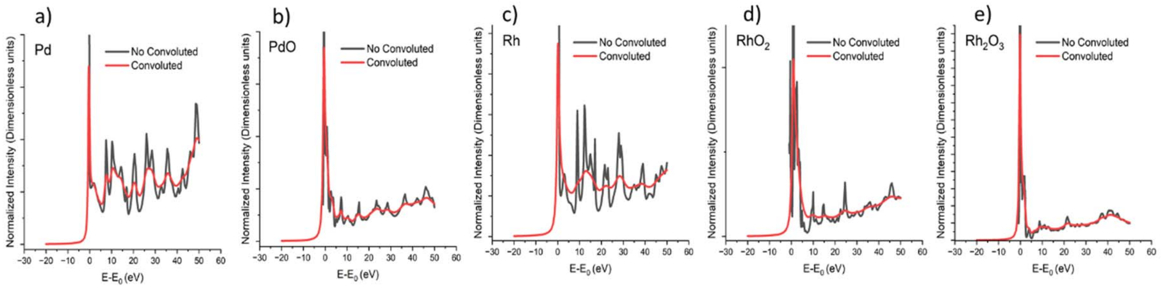

Figure S2 FDMNES simulations for Pd, PdO, RhO_2, and Rh_2O_3 with a radius of 9 Å. The red line shows convoluted spectra, and the dark line indicates non-convoluted spectra.

References

Joly, Y., Bunău, O., Lorenzo, J. E., Galera, R. M., Grenier, S. & Thompson, B. (2009). *J. Phys.: Conf. Ser.* **190**, 012007.

Svyazhin, A., Nalbandyan, V., Rovezzi, M., Chumakova, A., Detlefs, B., Guda, A. A. & Glatzel, P. (2021). *Inorg. Chem.*, **61(2)**, 869-881.

Amidani, L., Vaughan, G. B., Plakhova, T. V., Romanchuk, A. Y., Gerber, E., Svetogorov, R. & Kvashnina, K. O. (2021). *Chem. – Eur. J.*, **27(1)**, 252-263.
